# COVID-19 vaccination roll-outs in eleven small countries within the WHO European region; Lessons learned

**DOI:** 10.3389/fpubh.2022.959227

**Published:** 2022-08-18

**Authors:** Katie Palmer, Leda Nemer, Siddhartha Sankar Datta, Bettina Maria Menne

**Affiliations:** ^1^WHO European Office for Investment for Health and Development, Venice, Italy; ^2^WHO Regional Office for Europe, Copenhagen, Denmark

**Keywords:** COVID-19, WHO European Region, vaccines, immunization, prevention, vaccine strategies, SARS-CoV-2

## Abstract

The development and administration of COVID-19 vaccines has been an essential element in controlling the COVID-19 pandemic. However, countries worldwide have faced challenges in planning and implementing vaccination strategies. The aim of the current paper is to describe the situation faced by small countries in the WHO European Region in implementing their national vaccination strategies during the first stages of the planned roll-out (up to May 2021). This paper uses information from the WHO Small Countries Initiative (SCI), which includes a network of 11 countries with populations of ≤ 2 million (Andorra, Cyprus, Estonia, Iceland, Latvia, Luxembourg, Malta, Monaco, Montenegro, San Marino, and Slovenia). The SCI countries faced many challenges including: a lack of appropriate vaccination centers, adequate workforce, and registration/booking systems to cope with the unprecedented vaccine storage and administration demands; difficulties for high-risk groups (e.g., older individuals and those with health problems or cognitive impairment) to access vaccination sites or use digital registration/booking systems; vaccine wastage due to canceled appointments; and inequalities in vaccine uptake. Innovative programmatic interventions were implemented to facilitate the vaccination uptake of the populations such as: the creation of non-medical vaccination sites and mobile vaccination units; on-site vaccination of people in long-term residential facilities and long-term medical wards; diversifying health workforce like redeployment of healthcare professionals and use of medical students and retired medical professionals; campaigns with clear information to the general public (in multiple languages where necessary) both offline and online; use of digital registration/booking systems and alternative (non-digital) registration/booking systems for relevant individuals; and administration of excess vaccine doses to non-priority groups to avoid wastage.

## Introduction

### The public health impact of the COVID-19 pandemic

Since the start of the COVID-19 pandemic, there have been more than 516 million documented cases worldwide and over 6 million deaths with a large proportion in the WHO European region (217,646,171 confirmed cases and 1,999,825 reported deaths; data as of 13/05/2022)^1^. People infected with SARS-CoV-2 can present with a range of symptoms at different severity levels. In addition to the risk of serious illness and mortality, COVID-19 also poses potential long-term consequences for some individuals, such as prolonged respiratory, neurological, and cognitive symptoms (1–4). Alongside these risks, the health of population has been potentially affected as an indirect result of varied public health measures implemented by the countries to control the pandemic, such as social distancing and lock-downs, which have led to disruptions to routine medical care and management of non-communicable diseases, and increased risks for mental health (5–8).

### Vaccination strategies against COVID-19

Vaccination has been an essential strategy in controlling the COVID-19 pandemic. Since the first vaccine was approved for use in December 2020 by the European Medicines Agency^2^ over one billion doses (1,577,389,178; data as of 13/05/2022) have been administered in the WHO European Region^3^. The initial vaccination roll-out was focused on delivering complete primary vaccination series to health and social care professionals and individuals with underlying disease conditions, which progressively was extended to other non-priority population groups. As multiple COVID-19 vaccines became available at the end of 2020 and start of 2021, health systems worldwide struggled with varying challenges in implementing their vaccination strategies. Many countries continue to face repeated waves of COVID-19 and there have been ongoing fears of variants of concern; for example, in November 2021 the WHO designated Omicron as a variant of concern^4^ and, although little was known about the variant at that time, research subsequently showed that it is transmitted much more rapidly than other variants of SARS-CoV-2 (9). Further studies found that primary immunization with two doses of different vaccines (ChAdOx1 nCoV-19 or BNT162b2) provided some limited protection against symptomatic disease, whereas booster doses substantially increased protection, which then waned over time (10). These points underline the vital need to continue to plan and implement vaccinations with short- medium-, and, long-term perspectives. Each country faces different challenges; identifying these difficulties and sharing the methods they used to overcome them are essential. Countries with smaller populations might have specific characteristics that affect them, such as differences in infrastructures or flexibility within their health systems. Evaluating how such countries handled vaccination roll-outs and describing both positive and negative aspects of the strategies may help for future planning.

### Aims

The aim of the current paper is to outline the situation faced by small countries in the WHO European Region regarding their COVID-19 vaccination strategies during the first stages of the planned roll-outs (up to May 2021) using information from the WHO Small Countries Initiative (SCI), described later^5^. The overall objective is to provide a commentary on the data and information about vaccine roll-outs in the SCI countries and discuss the perspectives of people involved in the process, including the key barriers that prevented vaccines from being as widely and quickly administered as possible. Specifically, we aim to describe the challenges that SCI countries faced in procuring vaccines, setting up vaccination sites, and administrating vaccines to different priority groups; describe the systems used for vaccine registration and vaccination efforts for the general population as well as for vulnerable groups; and share the methods that the countries used to address problems. General challenges will be discussed as well as issues faced by specific countries. Of note, this report was first created as a snapshot to describe the initial roll-out strategies (from December 2020 to May 2021) and does not reflect any new approaches undertaken in the countries beyond May 2021 like booster vaccinations.

## Methods

### The Small Countries Initiative (SCI), WHO European Centre for Investment for Health and Development, Venice, Italy

The Small Countries Initiative (SCI)^5^ includes a network of 11 Member States in the WHO European Region with populations of 2 million or less, comprising of 6 countries in the European Union (EU), namely Cyprus^6^, Estonia^7^, Latvia^8^, Luxembourg^9^, Malta^10^, and Slovenia^11^ as well as 5 non-EU countries, namely Andorra, Iceland^12^, Monaco^13^, Montenegro^14^, and San Marino^15^. The Initiative was established in 2013 and the Secretariat is run from the WHO European Center for Investment for Health and Development, Venice, Italy. The objectives of this initiative are to leverage existing opportunities in small countries at the regional and international levels; share best practice in implementing relevant policies, strategies, and interventions; and build capacity in several high-priority thematic areas including practice-based evidence on ways to address different public health challenges. Within the context of the COVID-19 pandemic, the initiative has created an innovative platform for collaborative sharing of knowledge and experiences related to the pandemic, including those that may create specific and unique problems for countries with small populations, which differ considerably in land mass and population density (Table 1) as well as in terms of single country negotiation and purchasing power.

**Table 1:** Vaccine coverage (%) of the total population for small countries in the WHO European Region in week 21, May 2021 and week 18, May 2022.

	**Andorra**	**Cyprus**	**Estonia**	**Iceland**	**Latvia**	**Luxembourg**	**Malta**	**Monaco**	**Montenegro**	**San Marino**	**Slovenia**
Total population in thousands (2020)^*a*^	77	1,207	1,327	341	1,886	626	442	39	628	34	2,079
Population density per km^2^ (2020)^*a*^	164.4	130.7	31.3	3.4	30.3	241.7	1,379.8	26,338.3	46.7	565.6	103.2
**Vaccination coverage in epidemiological week 21 of May 2021** ^**b**^											
First dose uptake^d^	35.7%	40.7%	36.8%	45.1%	21.6%	36.4%	53.5%	43.3%^c^	16.9%	64.8%	31.8%
Complete series uptake^e^	10.8%	23.8%	18.3%	22.1%	9.0%	20.6%	35.6%	35.5%^c^	5.3%	58.9%	19.3%
**Vaccination coverage in epidemiological week 18 of May 2022** ^**f**^											
Uptake with at least one dose^*g*^	70.1%	74.7%	63.3%	84.1%	68.2%	74.9%	87.4%	59.8%	44.5%	76.5%	59.1%
Complete series uptake^h^	70.1%	74.3%	63.0%	81.4%	66.5%	73.8%	86.2%	59.5%	40.6%	68.6%	57.6%
Uptake with additional dose^***i***^	54.3%	52.0%	34.2%	68.5%	27.6%	57.5%	67.4%	29.2%		56.0%	30.9%

^a^Data from the Population Division of the Department of Economic and Social Affairs of the United Nations Secretariat. World Population Prospects 2019 (online database). New York: United Nations; 2019 (https://population.un.org/wpp/Download/Standard/Population). ^b^ Data officially reported to WHO for epidemiological week 21, 2021 as reported by 1st June 2021 (https://worldhealthorg.shinyapps.io/EURO_COVID-19_vaccine_monitor/). ^c^ Data reported directly from the Principality of Monaco. ^d^ Percent of total national population receiving the first dose series of any product administered by end of reporting week (week 21, 2021). ^e^Percent of total national population receiving a complete dose series of any product by end of reporting week (week 21, 2021). ^f^ Data officially reported to WHO for epidemiological week 18, 2022 as reported by May 10th 2022. ^g^ Percent of total national population receiving the first dose series of any product administered by end of reporting week (week 18, 2022). ^h^ Percent of total national population receiving a complete dose series of any product by end of reporting week (week 18, 2022). ^i^Additional dose refer to vaccine doses either administered as an extension of the primary series (e.g., in immunocompromised individuals) or as a booster dose in already fully vaccinated individuals, by end of reporting week (week 18, 2022).

### Data sources

More information on the SCI and the 11 countries can be found on the respective official sites^1^^–^^15^. Vaccine coverage data was collected from the WHO/Europe COVID-19 Vaccine Programme Monitor (see text footnote 3). We present vaccine coverage from two time points: (i) epidemiological week 21, 2021, to provide a snapshot of how each country progressed during the first months of roll-out and (ii) more recent data from epidemiological week 18 of May 2022. The data are shown as the percentage of the total national population and include: (i) the first dose series of any product or (ii) a complete primary dose series of any product (i.e., receiving two doses of a two-shot vaccine or only one dose in the case of single-shot vaccines). Although the vaccination plans for booster doses are not discussed in the current paper, Table 1 also presents recent data on the number of booster doses administered, defined as the “total number of doses administered as extension of primary series or as a booster dose.”

If not otherwise referenced, all other information comes from the COVID-19 Health System and Response Monitor^16^ (HSRM), national Ministry of Health COVID-19 websites for each country^17^ and interviews with country focal points in the SCI network up to 31 May 2021 *via* email correspondence and video conference calls, coordinated by two of the authors of the paper (LN and KP), see Table 2 for the structure of each survey. Data and information collected *via* the other sources were confirmed with each country focal point. There were different response rates from each country to the vaccination-related interviews and, hence, data and information completeness varies between countries.

**Table 2:** Questions used as a base for email and video-call interviews with country focal points in the Small Countries Initiative.

**General questions**
Does the country have a National Vaccination Deployment Plan, NVDP, and when was it issued? What strategies did you use for vaccine procurement (non-EU countries), e.g., bilateral agreements, direct procurement, COVAX etc. What specific challenges affected the vaccine plan in your country? Did your country have already-existing vaccination centers (and relevant staff) that could cope with the vaccination storage and administration needs? If not, what strategies were used to address this? What digital health systems and tools were used in your country for vaccination booking or providing information to residents? Was there a specific strategy for reaching out to vulnerable/cognitively impaired persons who have difficulties using digital booking systems? Is there a specific initiative to administer excess doses to groups not currently in the “priority” categories (i.e., groups that are not yet eligible for vaccination)? Do you have any data on specific groups who have a low vaccination uptake (ages, socioeconomic status, ethnicity etc)? Can you describe any specific methods that your country used to overcome challenges to the vaccination roll-out?

## Vaccination roll-outs in eleven countries in the SCI: Experiences, challenges, and solutions

### Vaccination coverage

Table 1 shows the percentage of the population in each country who had received at least one dose or had completed the primary dose series of any vaccine by the end of May 2021 (epidemiological week 21). More than half of the population of Malta and San Marino had already received their first vaccine dose, while in most of the other SCI countries over one third of the population had received their first dose. In April 2021 the European Technical Advisory Group of Experts on Immunization (ETAGE)^18^ recommended an interim vaccination uptake target of 80% of the adult population, as soon as possible. More recent data from May 2022 (epidemiological week 18, 2022) demonstrates progress in terms of vaccination uptake, with almost all countries reporting complete primary series uptake in more than half of the population. Most SCI countries have also started administering booster doses as extensions of primary series and coverage is already more than half in Andorra, Cyprus, Iceland, Luxembourg, Malta, and San Marino.

### Vaccine procurement

In the initial roll-out, one of the major challenges faced by the small countries were related to vaccine procurement. The 6 EU countries were part of the EU joint procurement of European Medicines Agency (EMA)-approved vaccines. The non-EU countries secured vaccines using different mechanisms. Iceland signed direct agreements with AstraZeneca, Janssen, Moderna, and Pfizer. Andorra used methods of procuring bilaterally (with France and Spain from their EU negotiations) and from COVAX (Vaccine pillar of the Access to COVID-19 Tools Accelerator), purchasing ChAdOx1-S (recombinant) vaccine against COVID-19 (AstraZeneca COVID-19 vaccine AZD1222) as a self-financing country. Montenegro procured through direct orders from vaccine manufacturers, through COVAX, and *via* bilateral and trilateral agreements with countries. San Marino's approach included a bilateral procurement with Italy, followed by a direct purchase of Sputnik V. As for San Marino, although the bilateral procurement related to all vaccines that were sent to Italy, it was agreed that only the Pfizer/BioNTech COVID-19 vaccine would be sent to San Marino by Italy. As the Sputnik V vaccine had not yet been approved by the EMA or the WHO Emergency Use Listing Procedure (EUL), in May 2021 there was uncertainty over vaccinating frontline workers who commute daily between Italy and San Marino in terms of which country's National Deployment and Vaccination Plan should be followed.

### Repurposing of existing facilities and staff for vaccine administration

Most countries lacked sufficient, appropriate vaccination centers to cope with the unprecedented demands and scale of vaccination, particularly in remote areas. The smallest countries (<100 km2), i.e., Monaco and San Marino, opened just one to two vaccination centers. Other countries temporarily boosted their capacity by repurposing other facilities as COVID-19 vaccination sites. All countries used vaccination centers, health centers, and other medical facilities as the sites to provide vaccination. Malta also created non-medical vaccination sites (such as university campuses, council offices, and military facilities) and Estonia offered vaccination in some workplaces and engaged mobile units. Due to a shortage of vaccination sites in some parts of Latvia, mobile vaccination units were deployed. Andorra required help from France and Spain to get sufficient amounts of vaccination materials.

Most countries, including Andorra, Cyprus, Iceland, Malta, and Latvia employed nurses and/or physicians to administer vaccines while Malta also involved medical, dental, pharmacy, and nursing students amongst others. San Marino engaged the support of retired medical staff to meet patients on arrival at the vaccination centers and process consent forms. Estonia created a free online training course for immunizers.

### Vaccination campaigns

Vaccination plans were largely supported with poster and digital campaigns targeted to the general population informing them of which groups were currently eligible for vaccination and to encouraging uptake. For example in Luxembourg^19^, clear campaign posters were created in multiple languages (Luxembourgish, French, German, English, and Portuguese), with specific information on the characteristics (age range, specific medical conditions, etc.) of people eligible for vaccination for each of the phases. Malta also had a broad communications strategy targeting the specific age groups through various media, and also pro-actively managed misinformation.

### Digital and non-digital registration and booking systems

Combinations of online and telephone registration and booking systems were used to facilitate the management of vaccine administration. By the end of May 2021, all countries had implemented some form of digital system for their vaccination services. These were used for registration, booking appointments, and/or joining waiting list *via* official websites, mobile apps, and telephone hotlines. Latvia^20^ and Estonia^21^ used interactive maps on their COVID-19 websites to enable individuals to search for the closest vaccination centers.

Initiatives were created to target high-risk and vulnerable groups who may have low digital health literacy or are unable to independently book their vaccinations, even by telephone, such as people with advanced age, serious illness, or cognitive impairment. For example, in Estonia, people in the priority group were contacted directly *via* their family physician. Andorra used records from local authorities to identify and contact people who were unable to independently book appointments. In Malta, persons in the high priority groups were sent appointment letters by post, whereas persons in the lower priority groups (i.e., <60 years) could register online or through a text messaging service. Latvia initiated a campaign of proactively calling individuals aged ≥60 years in less vaccinated areas of the country to inform them of the ongoing vaccination campaign and offer appointments.

In all countries, a major challenge was finding methods to vaccinate individuals with physical or health problems that limit their ability to reach vaccination sites. Individuals living in long-term care facilities or geriatric medical wards were usually vaccinated on-site by the institutions' staff or external healthcare providers, while people receiving medical care at home were vaccinated by mobile teams in Estonia, Luxembourg, Montenegro, and Malta.

### Prioritization for vaccination to specific population groups according to their risk of being infected with SARS-CoV-2 or increased risk of COVID-19 complications

In order to reap the benefits of the limited vaccine dose availability at the start of the vaccination roll-out and allowing the healthcare system to adapt to administering vaccines on a large scale, all SCI countries devised a clear list of priority population groups in their National Vaccine Deployment Plans, with phases identified according to the risk of the population groups to COVID-19 infection. These included older individuals and those living in medical or long-term care facilities, followed by people with underlying disease conditions that could put them at a higher risk of adverse COVID-19 outcomes. Alongside, healthcare and social care workers, especially those working with COVID-19 patients or within emergency medicine, were prioritized due to increased risks of being infected with SARS-CoV-2 and potentially transmitting the infection to vulnerable patients.

Iceland^22^, Andorra^23^, and San Marino^24^ included specific employment groups, such as emergency services, prison, and education staff in their priority listing based on their local epidemiological situation. San Marino and Iceland^25^, however, delayed vaccination of individuals with documented COVID-19 infection in the past 6 months while prioritizing others without any prior known infection.

Some countries lengthened the intervals between doses to aim for wider coverage of the population with a first dose. For example, in Luxembourg, the interval between the first and second doses of 4 weeks was changed to 8–12 weeks for the AstraZeneca vaccine and, in March 2021, the Estonian Immunoprophylaxis Expert Committee^25^ recommended extending the interval between the first and the second dose of the AstraZeneca vaccine from 8 weeks to 12 weeks, and the interval between the doses of the Pfizer/BioNTech vaccine to 6 weeks.

### Extending the reach of vaccines to non-priority populations with leftover doses

Initially, vaccines were targeted to the high-risk priority groups, but many countries subsequently introduced systems to allow individuals from non-priority groups to register an interest (usually digitally) in receiving excess vaccine doses (e.g., in Andorra, Estonia, Luxembourg, and San Marino). When individuals from a higher priority group were unavailable or canceled a booking in Latvia, vaccination staff were instructed to administer excess doses to people from lower priority groups. Usually, individuals needed to demonstrate that they were able to reach a vaccine site with short notice (e.g., volunteers in Luxembourg could register if they were able to arrive at a vaccination site within 20 mins). By the end of May 2021, San Marino began to offer vaccinations to tourists. In April 2021, nurses in Estonia who had been sent to vaccinate people at home because they were unable to leave their house for health reasons were authorized to also vaccinate any carers or other household members.

### Efforts to address low vaccine uptake

At the time of collecting information, the SCI countries had too few available data on the characteristics of people with low vaccine uptake but believed that there were cases of inequity in vaccine uptake due to vaccine hesitancy as well as differential access to information and vaccine sites. Estonia observed a particularly low uptake in older individuals in one county. In response, the authorities launched a “1 + 1 campaign”^26^ in April 2021, where younger persons would receive a vaccine if they accompanied an older relative, friend, or neighbor to the vaccine site (at that point in time, the younger age group were not eligible to receive vaccines). Latvia observed lower vaccination coverage amongst older individuals (over 88 years of age), possibly due to vaccine hesitancy and reduced access to information from national authorities. Montenegro reported that some regions had a lower coverage, but more research is needed to establish reasons underlying the low uptake.

## Discussion

The small countries in the WHO European region faced many challenges during the first 6 months of the COVID-19 vaccine roll-out. In addition to public health measures, vaccination has been an essential element to control the COVID-19 pandemic, and the subsequent introduction of vaccine certificates in the EU^27^ and elsewhere helped to open countries up and allow people to travel with reduced COVID-19 risks. Nevertheless, there are substantial challenges in terms of short-, medium-, and long-term planning. Most countries have now faced multiple waves of COVID-19 and several variants of concern have raised new threats. Many countries worldwide are now vaccinating younger age groups but in order to have a wider impact with the available vaccines, it is prudent that the focus should remain on the high-risk vulnerable population groups for both primary series and booster doses. The evolving nature of changes in strategies and target groups require immense planning and the current article may provide practical insight into how smaller countries could address potential issues in the future. Research is ongoing to assess the duration of immunity following vaccination, and their efficacy against variants of concern. In May 2021, Monaco's SCI country focal point reported that they had initiated a project to assess antibody levels in the general population post-vaccination (after 1, 6, 12, and 24 months), which may contribute to the ongoing body of evidence.

The strength of the current study is that we were able to interview country focal points *via* video conferencing to gain first-hand perspectives on examples of good practice as well as challenges in the vaccine roll-outs in the SCI countries. This, combined with details of specific aspects of the rolls-out and strategies undertaken by each country, provides a relevant description of the challenges that small countries faced during the initial stages, and these are in line with findings from other studies in the literature that have compared small states and islands in Europe. One paper comparing the impact of COVID-19 in six European microstates (Andorra, Liechtenstein, Malta, Monaco, San Marino, and Vatican City) discussed how vaccination roll-outs were key in containing the pandemic, and described how all countries except Malta (which benefitted from the EU joint procurement) had to find different ways to procure vaccines from adjacent nations or directly from manufacturers (11). Another article comparing different vaccination roll-out speeds between Cyprus and Malta (12) highlighted that, although the two islands share similar demographic characteristics, formed part of the EU advanced vaccination purchase agreement, and had similar vaccine strategies, there was a slower roll out in Cyprus that may have been due to vaccine hesitancy, differences in the vaccine registration systems, and the fact that individuals could choose which vaccine brand to take. Low vaccination hesitancy and high vaccine doses have been proposed as the major factors underlying Malta's rapid vaccination roll-out (13). However, despite this, Malta experienced higher COVID-19 infection and mortality rates during the second waves, compared to other European island states with small populations (Cyprus and Iceland), emphasizing that vaccine roll-out is not the only factor needed to contain the pandemic, as restrictive measures and governance approaches also play an important role (14).

Some limitations deserve mention. First, this paper provides a description of how the SCI countries rolled out their vaccines in the initial stages and does not reflect on the subsequent phases such as the planning for booster dose administration. The COVID-19 pandemic is a fast-changing situation, with new waves and variants emerging, and what we know about treatment, vaccination, prevention, and long-term effects is constantly changing. Therefore, this article should be looked at as snapshot of the initial stages to better understand how well small countries in the WHO European Region were able to cope and adapt to this unprecedented challenge.

In conjunction with planning the practical aspects of ongoing and future vaccination strategies, it is essential to develop interventions to increase vaccination uptake in specific population groups who are hesitant and to assess the effectiveness of such initiatives. Within the SCI, we further plan to establish whether there have been geographical limitations due to land mass or population density in the small countries, particularly any challenges related to urban vs. rural locations. It is also relevant to understand how effective digital tools have been in vaccination monitoring, allocation, and booking in the small countries as well as which population groups need alternative non-digital booking and monitoring systems and how they can be adapted to suit their needs. The SCI will continue to examine such questions during the upcoming months within the domain of the ongoing threat of the COVID-19 pandemic.

## Data availability statement

Publicly available datasets were analyzed in this study. This data can be found here: https://covid19.who.int/; https://worldhealthorg.shinyapps.io/EURO_COVID-19_vaccine_monitor/.

## Author contributions

KP, LN, SD, and BM defined the objectives. BM is the Coordinator of the Small Countries Initiative at the WHO European Office for Investment for Health and Development, Venice, Italy. KP conducted data searches on vaccination coverage, information on each country's vaccine planning and response, and drafted the manuscript. KP and LN interviewed country focal points to collect information on each country's vaccine roll-strategy. LN, SD, and BM critically revised the manuscript. All authors approved the final manuscript before submission.

## Conflict of interest

The authors declare that the research was conducted in the absence of any commercial or financial relationships that could be construed as a potential conflict of interest.

## Publisher's note

All claims expressed in this article are solely those of the authors and do not necessarily represent those of their affiliated organizations, or those of the publisher, the editors and the reviewers. Any product that may be evaluated in this article, or claim that may be made by its manufacturer, is not guaranteed or endorsed by the publisher.

## Author disclaimer

The authors alone are responsible for the views expressed in this publication and they do not necessarily represent the decisions or the stated policies of the World Health Organization.

## Footnotes

^1^ https://covid19.who.int/

^2^ https://www.ema.europa.eu/en/medicines/human/EPAR/comirnaty#authorisation-details-section

^3^ https://worldhealthorg.shinyapps.io/EURO_COVID-19_vaccine_monitor/

^4^ https://www.who.int/news/item/28-11-2021-update-on-omicron

^5^ https://www.who.int/europe/initiatives/small-countries-initiative

^6^ https://eurohealthobservatory.who.int/monitors/hsrm/hsrm-countries/hsrm/cyprus

^7^ https://eurohealthobservatory.who.int/monitors/hsrm/hsrm-countries/hsrm/estonia

^8^ https://eurohealthobservatory.who.int/monitors/hsrm/hsrm-countries/hsrm/latvia

^9^ https://eurohealthobservatory.who.int/monitors/hsrm/hsrm-countries/hsrm/luxembourg

^10^ https://eurohealthobservatory.who.int/monitors/hsrm/hsrm-countries/hsrm/malta

^11^ https://eurohealthobservatory.who.int/monitors/hsrm/hsrm-countries/hsrm/slovenia

^12^ https://eurohealthobservatory.who.int/monitors/hsrm/hsrm-countries/hsrm/iceland

^13^ https://eurohealthobservatory.who.int/monitors/hsrm/hsrm-countries/hsrm/monaco

^14^ https://eurohealthobservatory.who.int/monitors/hsrm/hsrm-countries/hsrm/montenegro

^15^ https://eurohealthobservatory.who.int/monitors/hsrm/hsrm-countries/hsrm/san-marino

^16^ https://www.covid19healthsystem.org/mainpage.aspx

^17^https://www.diariandorra.ad/noticies/nacional/2020/03/26/grafiques_del_covid_19_159015_1125.html, https://www.govern.ad/coronavirus, https://www.pio.gov.cy/coronavirus/eng/categories/vaccines-en, https://kkk.kriis.ee/en, https://vaktsineeri.ee/en/, https://www.covid.is/covid-19-vaccine, https://covid19.gov.lv/en, https://manavakcina.lv, https://covid19.public.lu/en/vaccination.html, https://deputyprimeminister.gov.mt/en/health-promotion/covid-19/Pages/landing-page.aspx, https://covid19.mc/en/fight-against-coronavirus/tout-sur-les-vaccins/, https://www.covidodgovor.me, https://vaccinocovid.iss.sm, https://www.gov.si/en/topics/coronavirus-disease-covid-19/

^18^ https://apps.who.int/iris/handle/10665/341095

^19^ https://covid19.public.lu/en/vaccination.html

^20^ https://app.powerbi.com/view?r=eyJrIjoiNjAyZGY5ZmQtMGRhYi00NmQ2LTliMTUtM2VkMjMxNmZmYjE4IiwidCI6ImRiYzkwMTJkLTYyOGItNDNkNC1iMTkwLThhNzMwZjdlMWU5NiIsImMiOjl9

^21^ https://vaktsineeri.ee/en/covid-19/getting-a-vaccination/

^22^ https://www.covid.is/covid-19-vaccine

^23^ https://www.bopa.ad/bopa/033018/Pagines/GD20210205_13_23_21.aspx

^24^ https://vaccinocovid.iss.sm/

^25^ https://www.sm.ee/en/news/immunoprophylaxis-expert-committee-updated-recommendations-using-covid-19-vaccines

^26^ https://news.err.ee/1608194392/1-1-vaccination-introduced-in-ida-viru-county-to-boost-coverage

^27^ https://www.europarl.europa.eu/news/en/press-room/20210604IPR05512/parliament-gives-its-final-green-light-to-the-eu-digital-covid-certificate
